# Supplementary material for: Biomarkers in the prediction of contrast media induced nephropathy – the BITCOIN study
Source: PLoS One. 2020 Jul 16;15(7):e0234921. doi: 10.1371/journal.pone.0234921 (PMC7365403; doi:10.1371/journal.pone.0234921)
Supplement: S1 Table — Univariate logistic regression for CI-AKI using the biomarkers urinary NGAL-, KIM-1- and calprotectin/creatinine as predictors in the overall study population and in the subgroup of subjects without overt CKD. The area under the curve (AUC) represents the discriminatory accuracy of each biomarker, as well as the concordance statistic for the regression model using eGFR, NGAL-, KIM-1- and calprotectin/creatinine ratios as independent variables. Moreover, the predictive value of the models of Inohara et al. and Ghani et al. are presented with and without including NGAL/creatinine ratio into the model. (DOCX) [file pone.0234921.s001.docx]

**Supplements**

**Table 1. Univariate and multivariable logistic regression model for CI-AKI.** Univariate logistic regression for CI-AKI using the biomarkers urinary NGAL-, KIM-1- and calprotectin/creatinine as predictors in the overall study population and in the subgroup of subjects without overt CKD. The area under the curve (AUC) represents the discriminatory accuracy of each biomarker, as well as the concordance statistic for the regression model using eGFR, NGAL-, KIM-1- and calprotectin/creatinine ratios as independent variables. Moreover, the predictive value of the models of Inohara et al. and Ghani et al. are presented with and without including NGAL/creatinine ratio into the model.

| Biomarkers | b | SE b | Coefficient (β) | | p | 95% CI | AUC (95% CI) |
| --- | --- | --- | --- | --- | --- | --- | --- |
| NGAL/creatinine  Overall study population  No overt CKD | 0.008  0.006 | 0.003  0.004 | 1.008  1.006 | | **0.005**  0.136 | 1.002 - 1.013  0.998 - 1.014 | 0.68 (0.60 - 0.81)  0.63 (0.45 - 0.80) |
| KIM-1/creatinine  Overall study population  No overt CKD | 0.001  0.001 | 0.001  0.001 | 1.000  1.000 | | 0.074  0.518 | 1.000 - 1.000  0.999 - 1.001 | 0.58 (0.46 - 0.70)  0.53 (0.37 - 0.69) |
| Calprotectin/creatinine  Overall study population  No overt CKD | 0.001  0.001 | 0.001  0.001 | 1.000  1.000 | | 0.822  0.756 | 1.000 - 1.000  1.000 - 1.000 | 0.54 (0.42 - 0.65)  0.54 (0.36 - 0.73) |
|  | | | | | | | |
| Prediction model | AUC (95% CI) | | | Prediction model | | | AUC (95% CI) |
| Overall study population | 0.68 (0.60 - 0.81) | | | No overt CKD | | | 0.62 (0.46 - 0.77) |
| Inohara et al. model | 0.68 (0.60 - 0.76) | | | Ghani et al. model | | | 0.57 (0.46 - 0.67) |
| Inohara et al. model + NGAL ratio | 0.73 (0.63 - 0.82) | | | Ghani et al. model + NGAL ratio | | | 0.69 (0.58 - 0.80) |
